# Supplementary material for: Resistance associated metabolite profiling of Aspergillus leaf spot in cotton through non-targeted metabolomics
Source: PLoS One. 2020 Feb 12;15(2):e0228675. doi: 10.1371/journal.pone.0228675 (PMC7015376; doi:10.1371/journal.pone.0228675)
Supplement: Attachment — Submitted filename: Comments PLOS.doc [file pone.0228675.s003.doc]

To be accepted for publication in PLOS ONE, research articles must satisfy the following criteria:

1. The study presents the results of original research. **The research seems to be original but of not of very practical use. There is no literature available on the incidence and the losses caused due to A. tubigensis anywhere on cotton crop.**

2. Results reported have not been published elsewhere. **Need to be checked online.**

3. Experiments, statistics, and other analyses are performed to a high technical standard and are described in sufficient detail. : **There is no information about the pathogen and identification/registration. There is no detail available wrt purity of seed and the authorized source. Methodology followed for the experimentation and inoculation is not clear; There is no reference for resistant (Sadori) and susceptible variety (CIM 88 573). How and on what basis these were selected before conducting the experiment on *Gossypium hirsutum. There are no clear cut details and or references about disease symptoms, ratting and* AUDPC estimation.**

4. Conclusions are presented in an appropriate fashion and are supported by the data. **The nontargeted metabolomics approach aims to study both known and unknown metabolites. To comprehend the huge chunks of data this may yield, one must couple it with advanced chemometric methods such as multivariate analysis, so that these can be grouped into smaller manageable chunks. In this article no clear cut and pin pointed conclusions are drawn and the chemicals detected in resistant variety without and with inoculation are mostly of known nature. The discussion and conclusion are very vague and not very relevant to the findings.**

5. The article is presented in an intelligible fashion and is written in standard English. **Need English editing, and reference write-up under discussion part need to be shortened. Too much detail is given in the discussion part. Relevant references particularly on *G. hirsutum* are missing. Reference arrangement and style is not corrects.**

6. The research meets all applicable standards for the ethics of experimentation and research integrity. **Not sure. To be checked by the editor.**

7. The article adheres to appropriate reporting guidelines and community standards for data availability. **Not sure. To be checked by the editor**
