# Supplementary material for: Resistance associated metabolite profiling of Aspergillus leaf spot in cotton through non-targeted metabolomics
Source: PLoS One. 2020 Feb 12;15(2):e0228675. doi: 10.1371/journal.pone.0228675 (PMC7015376; doi:10.1371/journal.pone.0228675)
Supplement: Attachment — Submitted filename: Answer to reviewer comments.doc [file pone.0228675.s004.doc]

**ANSWERS TO REVIEWERS COMMENTS**

**Resistance Associated Metabolite Profiling of Aspergillus Leaf Spot in Cotton through Non-targeted Metabolomics**

To be accepted for publication in PLOS ONE, research articles must satisfy the following criteria:

1. The study presents the results of original research. **The research seems to be original but of not of very practical use. There is no literature available on the incidence and the losses caused due to A. tubigensis anywhere on cotton crop.**

**Answer:**

In last two seasons, leaf spot disease has become one of the devastating cotton diseases in Pakistan and it was reported in national print newspapers in URDU language. No one reported this disease yet and after intensive field surveys and repeated pathogenicity tests, our lab has already reported these losses in an international journal. Our manuscript has been accepted and will be available online, very soon.

2. Results reported have not been published elsewhere. **Need to be checked online.**

**Answer:**

We do hereby declare that the results of this study have not been submitted or published, anywhere else.

3. Experiments, statistics, and other analyses are performed to a high technical standard and are described in sufficient detail. : **There is no information about the pathogen and identification/registration. There is no detail available wrt purity of seed and the authorized source. Methodology followed for the experimentation and inoculation is not clear; There is no reference for resistant (Sadori) and susceptible variety (CIM 573). How and on what basis these were selected before conducting the experiment on *Gossypium hirsutum.* There are no clear cut details and or references about disease symptoms, ratting and AUDPC estimation.**

**Answer:**

As mentioned in previous query, pathogen identification and pathogenicity results will be published online, soon. All the seeds were collected from authorized research institute of cotton and we have mentioned it in the text. Methodology followed for the experimentation and inoculation has been described in a detailed manner. References for resistant (Sadori) and susceptible variety (CIM 573) have been incorporated. Clear cut details about disease symptoms, ratting and AUDPC estimation have been added.

4. Conclusions are presented in an appropriate fashion and are supported by the data. **The nontargeted metabolomics approach aims to study both known and unknown metabolites. To comprehend the huge chunks of data this may yield, one must couple it with advanced chemometric methods such as multivariate analysis, so that these can be grouped into smaller manageable chunks. In this article no clear cut and pin pointed conclusions are drawn and the chemicals detected in resistant variety without and with inoculation are mostly of known nature. The discussion and conclusion are very vague and not very relevant to the findings.**

**Answer:** In the revised manuscript, both multivariate analysis and PLSDA have been performed and described. As per instruction, conclusion has been modified as a precise and informative finding for the reader. This study is of significant importance because the reaction of Cotton against *Aspergillus tubingensis* has never been elaborated before. Both discussion and conclusion have been modified, as per instruction.

5. The article is presented in an intelligible fashion and is written in standard English. **Need English editing, and reference write-up under discussion part need to be shortened. Too much detail is given in the discussion part. Relevant references particularly on *G. hirsutum* are missing. Reference arrangement and style is not corrects.**

**Answer:** English editing has been done carefully. Discussion has been revised and shortened to half. References relevant to *G. hirsutum* have been incorporated. Reference arrangement and style has been corrected.

**ANSWERS TO EDITOR’S COMMENTS**

**Resistance Associated Metabolite Profiling of Aspergillus Leaf Spot in Cotton through Non-targeted Metabolomics**

Reviewer Comments to the author:

Reviewer #1:

Query 1:

Line 18. The “and abiotic” should be deleted

Answer: Desired changes have been incorporated.

Query 2:

Line 19. The “behavior” is more relevant term to animals, rather than plants.

Answer: Plant relevant term “response” has been added.

Query 3:

Line 36. The level is same or different?

Answer: For better understanding, sentence has been reconstructed.

Query 4:

Line 39. Full stop should be added at the end of the sentence.

Answer: Required changes have been incorporated.

Query 5:

Line 45-53. The authors should focus more on Leaf spot of cotton (*Gossypium hirsutum* L.) and *Aspergillus tubingensis* rather than other pathogens of cotton

Answer: Literature related to *Gossypium hirsutum* and *Aspergillus tubingensis* has been added.

Query 6:

Line 69-70. Is this sentence in close relationship with the context?

Answer: As per instruction, desired changes have been made.

Query 7:

Line 98-100. The description is unclear, should be explained in a bit detail.

Answer: Fungal Inoculation methodology has been explained in detail.

Query 8:

Line 194-200 and Fig. 1 and 2. There are four treatment groups, including RT, RM, SM and ST. The authors have mentioned just two groups in Fig. 1 and 2. They should also include the other two groups. Meanwhile, the leaf spot area in susceptible and resistant varieties is much larger than 5 fold in Fig. 1, while in Fig. 2 it is shown about 5 fold. The ratio in both figures does not match.

Answer: Keeping in view the reviewer’s concerns, pictures of RM and SM have been added. Required changes have been incorporated.

Query 9:

Line 337. Reference [23] should be replaced with “Levi et al.” or the sentence should be rephrased and the reference should be placed at the end of the sentence.

Answer: Desired changes have been incorporated.

Query 10:

Line 382. “are said to” is very colloquial, so rephrase the sentence.

Answer: Desired changes have been incorporated.

Following all queries and suggestions, we have modified article. Kindly see changes and review at your earliest.

Regards,

Dr. Muhammad Farooq Hussain Munis

(CorrespondSing Author)
